# The impact of cell culture media on the interaction of biopolymer-functionalized gold nanoparticles with cells: mechanical and toxicological properties

**DOI:** 10.1038/s41598-022-20691-w

**Published:** 2022-10-05

**Authors:** Brahmaiah Meesaragandla, Yesaswini Komaragiri, Rabea Schlüter, Oliver Otto, Mihaela Delcea

**Affiliations:** 1 Biophysical Chemistry, Institute of Biochemistry, University of Greifswald, Felix-Hausdorff-Straße 4, 17489 Greifswald, Germany; 2 ZIK HIKE-Zentrum Für Innovationskompetenz „Humorale Immunreaktionen Bei Kardiovaskulären Erkrankungen“, Fleischmannstraße 42, 17489 Greifswald, Germany; 3 DZHK (Deutsches Zentrum für Herz-Kreislauf-Forschung), Partner Site, Greifswald, Germany; 4 Institute of Physics, University of Greifswald, Felix-Hausdorff-Strasse 6, 17489 Greifswald, Germany; 5 Imaging Center of the Department of Biology, University of Greifswald, Friedrich-Ludwig-Jahn-Str. 15, 17489 Greifswald, Germany

**Keywords:** Nanoparticles, Biomaterials - proteins

## Abstract

Understanding the nanoparticle-cell interactions in physiological media is vital in determining the biological fate of the nanoparticles (NPs). These interactions depend on the physicochemical properties of the NPs and their colloidal behavior in cell culture media (CCM). Furthermore, the impact of the bioconjugates made by nanoparticle with proteins from CCM on the mechanical properties of cells upon interaction is unknown. Here, we analyzed the time dependent stability of gold nanoparticles (AuNPs) functionalized with citrate, dextran-10, dextrin and chitosan polymers in protein poor- and protein rich CCM. Further, we implemented the high-throughput technology real-time deformability cytometry (RT-DC) to investigate the impact of AuNP-bioconjugates on the cell mechanics of HL60 suspension cells. We found that dextrin-AuNPs form stable bioconjugates in both CCM and have a little impact on cell mechanics, ROS production and cell viability. In contrast, positively charged chitosan-AuNPs were observed to form spherical and non-spherical aggregated conjugates in both CCM and to induce increased cytotoxicity. Citrate- and dextran-10-AuNPs formed spherical and non-spherical aggregated conjugates in protein rich- and protein poor CCM and induced at short incubation times cell stiffening. We anticipate based on our results that dextrin-AuNPs can be used for therapeutic purposes as they show lower cytotoxicity and insignificant changes in cell physiology.

## Introduction

Over the last few years, nanotechnologies have undergone an exponential growth in various fields such as nanomedicine, electronics, energy and environment due to the unique chemical and physical properties of the nanomaterials^1–3^. Metal nanoparticles such as gold nanoparticles (AuNPs) have attracted an increasing attention due to their wide applications e.g., in diagnostics, as drug carriers, for imaging and therapy^4–8^. This is due to their specific physico-chemical properties such as small size, greater surface area-to-volume ratio, high reactivity and surface plasmon resonance (SPR) which depend on particle size, shape and dielectric constant of the medium^9–14^. In order to design AuNPs as drug carriers or therapeutic agents it is necessary to investigate the interaction of NPs with cells, including their biological and biophysical implications.

When NPs are exposed to biological systems, they rapidly adsorb biomolecules onto their surface, potentially changing the equilibrium and leading to formation of various arrangements of NP-biomolecule complexes. The formation of NP-biomolecule complexes can vary with time and is an important aspect of assessing the impact of NPs on organisms^15,16^. In addition to proteins, biological fluids contain lipids, electrolytes, carbohydrates, amino acids and metabolites which might disturb the NPs’ environment and therefore, their colloidal stability. Mixing of these two independently stable systems (i.e. colloidal NPs and biological fluids) can result in the formation of various arrangements of NP-biomolecule complexes^17^. Such complexes have a different identity compared to the naked NPs and dictate the behaviour and fate of the NPs in biological systems including their agglomeration tendency, NP adhesion to the cell membrane, cell-uptake and possible toxicity^18^. For example, larger sized AuNPs or their aggregates in cell culture medium exhibit an increased sedimentation rate leading to higher uptake rates and increased toxicity^19^.

After interacting with cells, NPs can induce a series of typical changes in cell growth, differentiation, migration, and cytokine secretion, which play significant roles in physiological metabolism^20^. The interactions between NPs and cells can be affected by several factors, such as cell type, fluid environment and specific physicochemical properties. Researchers have focused on development of various approaches for particle design and functionalization, and the study of NP-protein interactions and their role on the NP cell uptake and toxicity^21–27^. A very few studies have focused on understanding the effect of NPs on cell mechanics, i.e., to study how mechanical stress impacts on the cytoskeleton exposed to NPs and how this relates to cellular function^28^. However, it is very important to study the in vitro and in vivo behaviour of NPs in complex biological systems and their effect on cell mechanics and cell viability because the NP-biomolecular complexes change with time.

It has been shown that the degree of internalization of AuNPs and their cytotoxicity depends on the size, morphology and charge of NPs^29,30^. For example, Zhang et al. demonstrated that the distribution and toxicity of AuNPs depend on the size of the NPs based on a study conducted on mice^31^. Positively charged NPs are more toxic compared to negatively charged or neutral NPs because the negatively charged cell membrane can attract positively charged NPs^32–36^. Albanese et al. have assessed the aggregation of AuNPs by sodium chloride and the effect of transferrin coating in cell uptake depending on the cell type, mechanism of uptake, and receptor expression^37^. In another study, Ha et al. studied the role of diffusion and sedimentation of silver NPs in upright- and inverted cell culture and found that NP stability is highly relevant to the effective dose, rather than properties of the bare particles^38^.

In order to make an objective and relevant comparison between cell response and toxicity, it is essential to characterize the NPs and their complexes in cell culture medium (CCM) at conditions similar to those in cell assays. Also, the impact of NPs on cell mechanics has been less thoroughly investigated compared to the cell viability studies. Therefore, an understanding of any possible alteration of cell mechanics following NP exposure with time is needed to enhance the comprehension of nanomaterial-cell interactions.

Here, we explore the effect of AuNPs surface functionalization on the mechanical properties and the viability of HL60 cells in medium supplemented with and without 10% fetal calf serum (FCS) protein. HL60 cell line is an attractive model for differentiation studies. HL60 cells grow in suspension culture and have the capability of differentiating into monocytes or macrophages upon induction with different chemical compounds such as dimethyl sulphoxide^39^. The behaviour of citrate-, dextran-10-, dextrin- and chitosan-functionalized AuNPs in both media (with and without FCS protein) at different time intervals has been investigated. The protein rich media chosen was the widely used and well-described CCM RPMI-1640, supplemented with FCS, whereas protein poor media is without FCS. Cell mechanical characterization has been performed at different time intervals using the high-throughput technology real-time deformability cytometry (RT-DC)^40–42^. Next, we evaluated the cytotoxicity of such AuNP-bioconjugates in both media. Finally, release of reactive oxygen species induced by the AuNPs in protein rich- and protein poor CCM in cells was studied.

## Results and discussion

### Characterization of functionalized AuNPs

Different surface-functionalized AuNPs were synthesized according to the method described in our previous paper^43,44^. AuNPs were functionalized with four ligands; citrate, dextran-10, dextrin and chitosan. The molecular structures of the used ligands are shown in Fig. S1. It is known that except citrate, all other ligands show low toxicity, high dispersibility and enhanced biocompatibility. Dextran-10 (dex-10) molecule is a branched molecule, whereas dextrin and chitosan molecules are linear in nature. All AuNPs types were well dispersed in water and showed the SPR peak at ∼ 528 nm (Fig. S2). An additional shoulder at 600 nm for dex-10-AuNPs is either due to the formation of some aggregates or due to formation of networks via the branched dex-10 molecules. TEM micrographs show that all types of AuNPs are spherical with an average diameter for citrate-, dex-10-, chitosan- and dextrin-AuNPs of 15.6 ± 1 nm, 13 ± 2 nm, 23.5 ± 6 nm and 6.4 ± 2.5 nm, respectively (Fig. S3).

The average hydrodynamic diameter (d_H_) for citrate-, dex-10-, chitosan- and dextrin-AuNPs determined by dynamic light scattering (DLS) in water was ∼ 19.2, ∼ 60, ∼ 64 and ∼ 40 nm, respectively (Fig. S4A). The overall increase in diameter compared to TEM data is due to the hydrated expansion of the molecules in aqueous state. The zeta potential of functionalized AuNPs was measured (Fig. S4B) to confirm the surface-functionalization. With the exception of chitosan-AuNPs which are positively charged due to the –NH_2_ groups, the surface of the other types of AuNPs was negatively charged due to the presence of –COOH and –OH groups.

### Time-dependent absorption behavior of AuNPs in protein poor- and protein rich cell culture media

To understand the interaction of AuNPs with components of CCM, we exposed different surface- functionalized AuNPs to CCM composed of RPMI supplemented with 10% FCS and without FCS at 37 °C for different time intervals. RPMI medium supplemented with 10% FCS is considered as protein rich CCM (= 7 mg/mL proteins), whereas RPMI without FCS considered as protein poor CCM. Protein poor CCM contains only proteins at very low concentration (ng/mL) in the form of growth factors. However, both CCM contain a high amount of amino acids, carbohydrates, vitamins and salts which can cover the surface of AuNPs.

**Figure 1:**
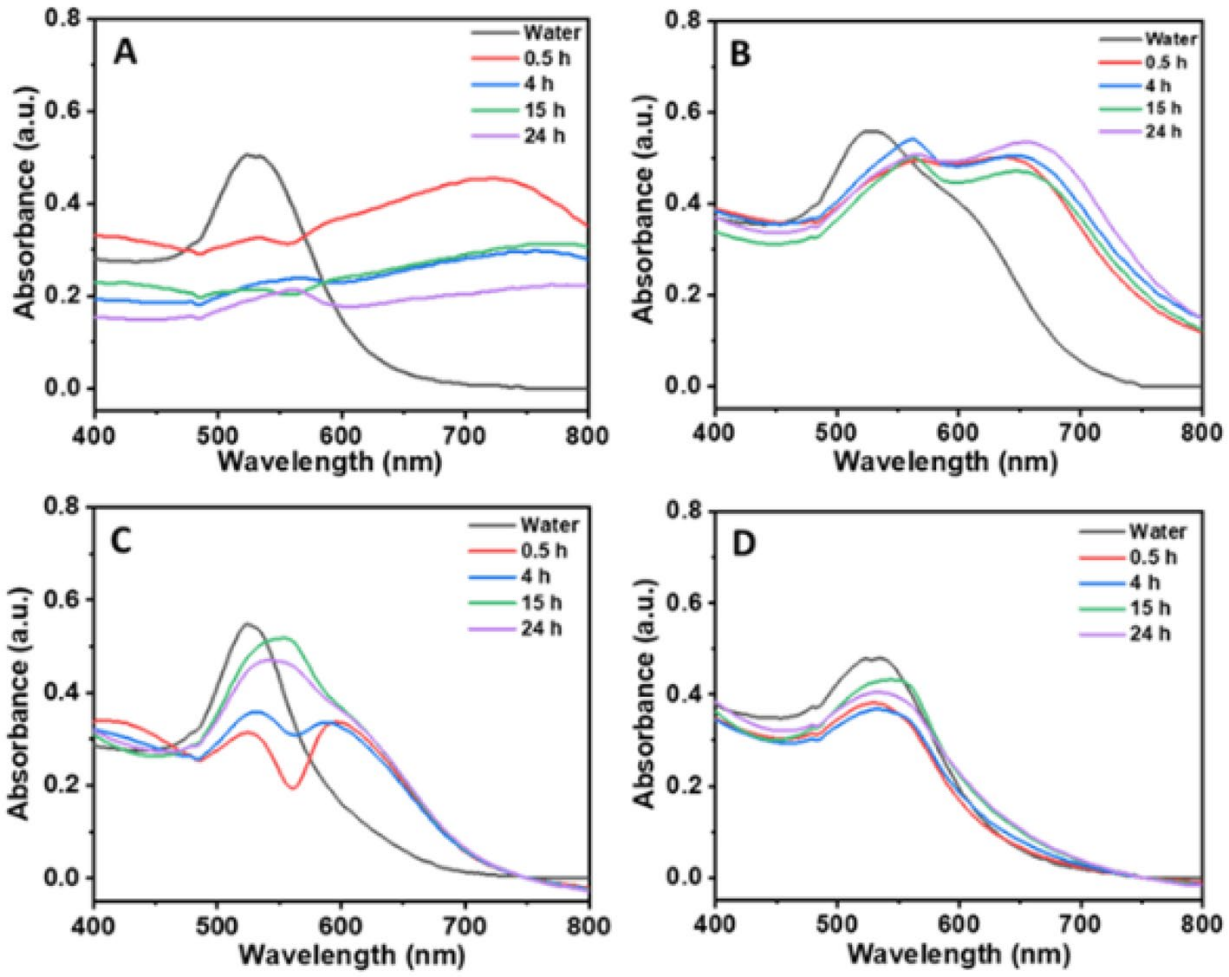
Time dependent UV–Vis spectra of (**A**) citrate, (**B**) dex-10, (**C**) chitosan and (**D**) dextrin-functionalized AuNPs in protein poor cell culture media at 37 °C. (AuNPs concentration = 100 nM).

First, we characterized the stability of the AuNPs over time in both protein poor- and protein rich CCM using UV–Vis spectroscopy. Figure 1 shows the UV–Vis spectra of the various surface-functionalized AuNPs in protein poor media at 37 °C for different time intervals (0.5, 4, 15 and 24 h). It can be seen that the SPR band of citrate-AuNPs shifted from 528 nm to longer wavelength 720 nm and the intensity was completely diminished with increasing time. Therefore, in the absence of serum proteins, the SPR band of the citrate-AuNPs rapidly disappears, redshifts, and broadens, and a new band at 550 and 750 nm emerges, which indicates the formation of larger non-spherical aggregates^45,46^. This is because citrate molecules are easily replaced allowing a spontaneous adsorption of proteins when they are exposed to high concentration of charged molecules in CCM due to its weak binding nature. Similarly, dex-10-AuNPs also shifts the SPR band from 528 to 560 nm with an additional band at 655 nm and suggests the formation of both spherical and non-spherical aggregates. However, no much change in the intensity of absorbance with time was observed in case of dex-10-AuNPs.

Although dex-10 molecules bind to the AuNPs strong enough compared to the citrate molecules, they also form aggregates due to branching nature of dex-10 molecules; which allows fewer binding sites on the AuNPs surface. In the case of chitosan-AuNPs, an extra band at 590 nm was observed along with the SPR band at 528 nm until 4 h incubation, suggesting the formation of spherical aggregates along with stable AuNP- bimolecular corona.

After 15 h incubation, the SPR band becomes broad with a peak maximum at 545 nm, which might be due to the formation of both stable AuNP-biomolecular conjugates and spherical aggregates. In case of dextrin-AuNPs, there was no shift in the SPR band until 4 h incubation, which indicates the formation of stable AuNP-biomolecular corona. However, after 15 h and 24 h of incubation, there was a slight shift in the SPR band from 528 to 535 nm suggesting strong interactions of AuNPs with biomolecules by the exchange of new molecules on the AuNP surface.

**Figure 2:**
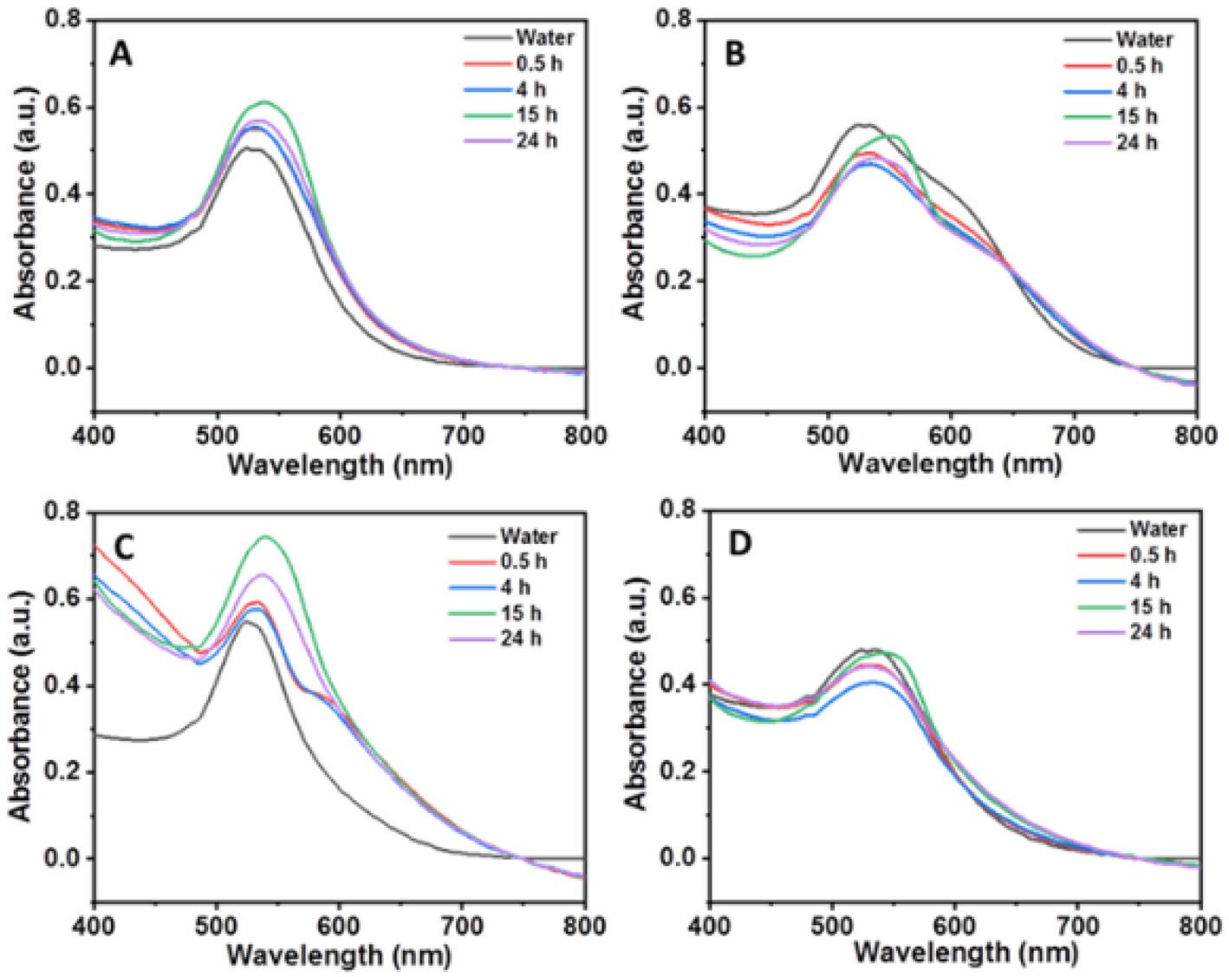
Time dependent UV–Vis spectra of (**A**) citrate, (**B**) dex-10, (**C**) chitosan and (**D**) dextrin-functionalized AuNPs in protein rich media at 37 °C. (AuNPs concentration = 100 nM).

Figure 2 shows the UV–Vis spectra of AuNPs in protein rich media at different time intervals (0.5, 4, 15 and 24 h). Except chitosan-AuNPs, all other AuNPs showed no shift in the SPR band up to 4 h incubation. Moreover, chitosan-AuNPs showed an additional band at 600 nm for 0.5 h and 4 h incubation. This might be due to the formation of some spherical aggregates by the strong electrostatic interaction between positively charged chitosan molecules and negatively charged proteins (e.g. serum albumin). However, after 15 h and 24 h incubation, a slight red shift in the SPR band was observed, which reveals strong interaction of all the AuNPs with biomolecules and formation of stable AuNP-biomolecular conjugates either by replacement or adsorption of biomolecules with time. It is important to consider that the aggregation of AuNPs is dependent on either direct contact between the surfaces of two AuNPs or weekly bounded ligands on the AuNPs surface and AuNP charge.

UV–Vis results suggest that the behavior of AuNPs changes with protein rich- and protein poor CCM. Dextrin-, and chitosan-AuNPs showed similar behavior in both protein rich- and protein poor CCM, whereas citrate- and dex-10-AuNPs change with media. We believe that AuNPs with weak ligands (citrate-, dex-10) undergo aggregation in protein poor media, whereas form stable conjugates in protein rich media. However, all the AuNPs changed their surface with time in both media as confirmed by shift in the SPR band with different incubation times. Similar behavior was observed in the past work for the magnetic nanoparticles, where an increased amount of serum proteins adsorbed on the NPs after 16 h of incubation^47^.

### Time-dependent size distribution of AuNPs in protein poor- and protein rich cell culture media

**Figure 3:**
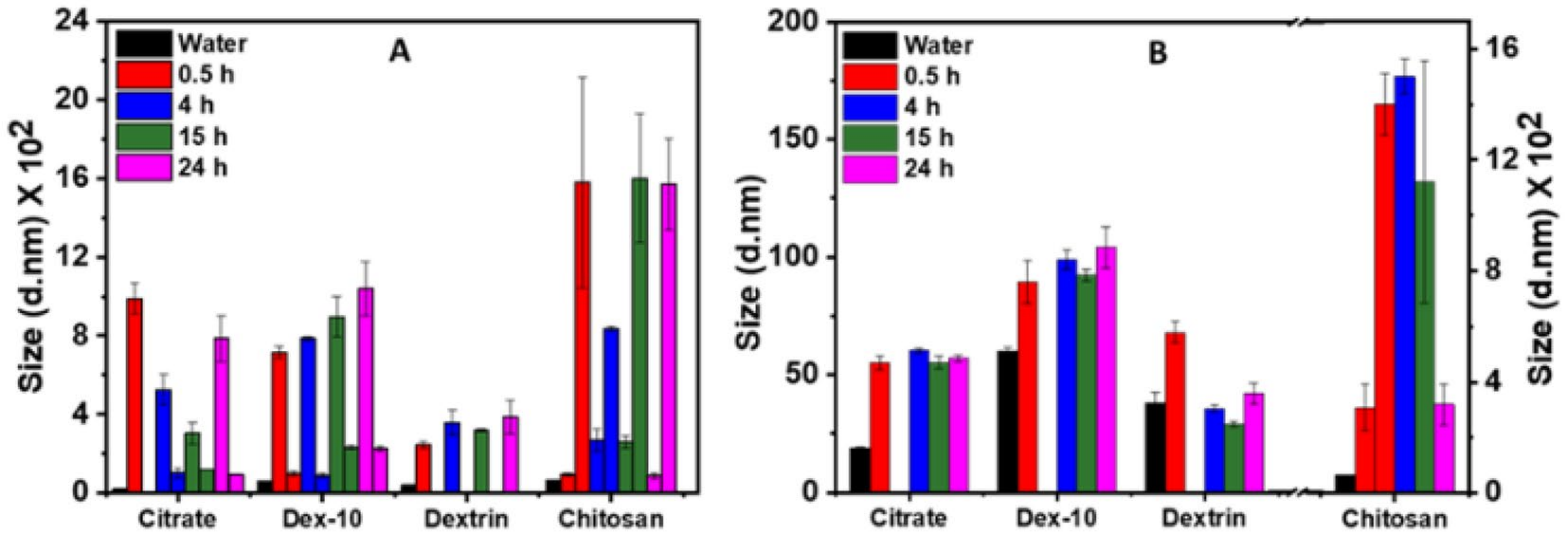
Time dependent DLS data of different surface-functionalized AuNPs in (**A**) protein poor media and (**B**) protein rich media at 37 °C.

To further understand the size and charge distribution of AuNPs in both protein poor- and rich media, we have carried out DLS and zeta potential measurements at different time incubations (0.5, 4, 15 and 24 h) at 37 °C. Figure 3A,B show the d_H_ of various surface-functionalized AuNPs in protein poor- and rich media at different time intervals. In case of protein poor media, except dextrin-AuNPs, all other AuNPs showed two different sizes, which indicate the formation of spherical and non-spherical aggregates. Both citrate- and dex-10-AuNPs displayed a d_H_ around 100 nm and 700–1000 nm; representing the formation of spherical and non-spherical aggregates. The variation in the d_H_ over time suggests a change in adsorption behavior of biomolecules on the AuNPs surface. However, chitosan-AuNPs, showed d_H_ around 100 nm and along with diameter around 1500 nm, which might be due to the formation of stable chitosan-AuNPs-biomolecular corona and non-spherical aggregates, respectively. Dextrin-AuNPs showed only a d_H_ around 250 nm, which is due to the formation of stable dextrin-AuNPs-biomolecular conjugates. In case of protein rich medium shown in Fig. 3B; except chitosan-AuNPs, all other AuNPs showed only one d_H_ around 40–100 nm, which indicate the formation of stable AuNP-biomolecular conjugates. Chitosan-AuNPs exhibit two peaks around 300 nm and 1500 nm which are altered with time, indicating the formation of stable and spherical aggregates. DLS data completely agrees with the UV–Vis spectra analyses.

To further understand the surface charge distribution of AuNPs in both protein poor- and rich media, we have studied the zeta potential of AuNPs after incubation at 37 °C for 24 h. Figure S5 shows the surface charge distribution of different surface-functionalized AuNPs in protein poor- and rich media at 37 °C after 24 h incubation. The surface charge of the AuNPs after incubation in both media showed increased negative values which also indicate the formation of a biomolecular corona.

Additionally, we have investigated the osmolarity of CCM in order to understand any change in the concentration of biomolecules by the addition of different surface-functionalized AuNPs with time. Generally, CCM is designed to have osmolarity between 260 and 320 milliosmoles (mOsm) to mimic the osmolarity of serum (290 mOsm/kg)^48^. Figure S6 shows the osmolarity of both protein poor- and rich CCM after incubation with the AuNPs for 0.5 and 24 h at 37 °C. Except dex-10-AuNPs, all other AuNPs maintained the osmolarity in both protein poor- and rich CCM. Dex-10-AuNPs in protein poor CCM exhibited increased osmolarity at shorter incubation time (0.5 h). However, after 24 h incubation there was no much change in the osmolarity. The increased osmolarity in protein poor CCM might be due to the change in the concentration of molecules by release of some dex-10 molecules. This data clearly shows that except dex-10-AuNPs, all the AuNPs have no influence on the concentrations of biomolecules in both CCM.

### Mechanical behavior of HL60 cells after treatment with AuNPs at different time intervals

We next studied the mechanical properties of HL60 cells after interaction with different surface-functionalized AuNPs at different incubation times. Cell elasticity can be used to express the resistance of the cell to an externally induced deformation. It is usually referred to as the Young’s modulus (E), which is the ratio between the applied mechanical stress and the resulting strain^49^. We have used the nomination Young’s modulus (instead of elastic modulus) as we are considering the cell as an homogeneous linear elastic material. In general, mechanical properties of a cell are related to the structure of the cytoskeleton. Alterations in mechanical properties can reflect changes in the cytoskeleton and by that in function. For example, actin microfilaments could be structured differently in healthy cells compared to unhealthy cells resulting in differences in the Young’s modulus^50^. However, these microfilaments are not well organized or are less observed in cancerous cells; hence the cells are softer and more flexible^51,52^.

Typically, cell stiffness is measured through indentation experiments using atomic force microscopy (AFM)^28^. AFM is a state-of-the-art surface sensitive method that has recently been used for understanding the nanoparticle-to-nanoparticle and cell interactions in physiologic fluids^53,54^. However, the application of AFM to study the mechanical properties of suspended cells is challenging because this method always requires the adherence of a sample to a surface.

One of the recent advancements in studying the biomechanics of cells is RT-DC, a technique which facilitates high throughput characterization of cells in suspension^40–42^. RT-DC being a microfluidic technique deforms the cells hydrodynamically and enables analysis in real-time at high speed (1000 cells s^−1^).

**Figure 4:**
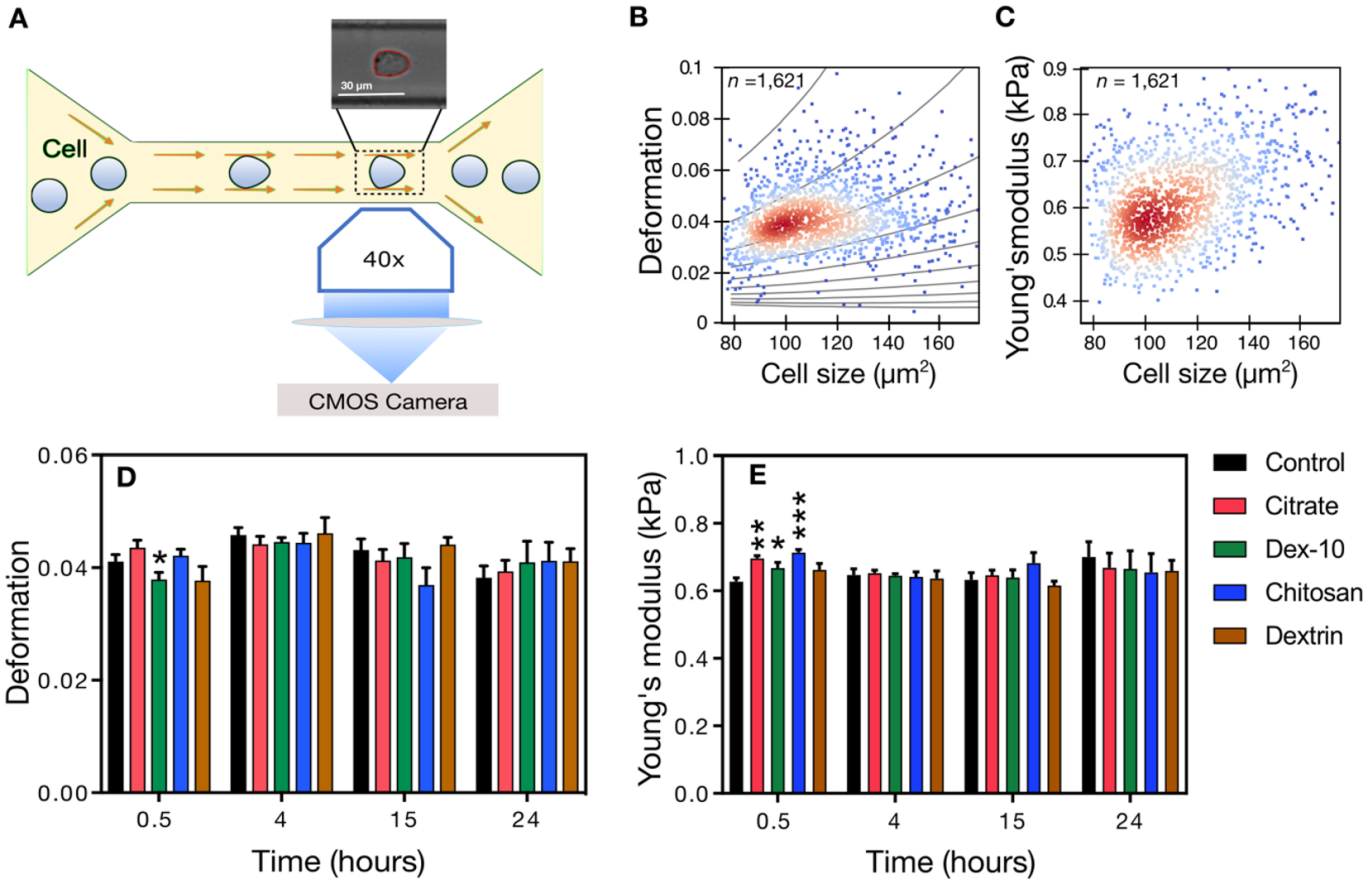
Time dependent mechanical alterations of HL60 cells in presence of different surface-functionalized AuNPs (50 pM) in RPMI without FCS. (**A**) Schematic representation of a microfluidic chip, where cells are deformed hydrodynamically by a parabolic flow profile at a flow rate 0.16 µls^−1^. Deformation and cell size is obtained from bright field images (top) captured by a 40× objective and CMOS camera using a contour (red) representing cell perimeter and area. (**B**) Scatter plot of deformation *vs.* cell size. Curved lines indicate the isoelasticity lines to calculate the Young’s modulus of the cells. (**C**) Scatter plot of Young’s modulus *vs.* cell size. Control cells (**B**, **C**) were measured after treatment with water. Color code (**B**, **C**) highlights event density, where red means highest density of the population. (**D**, **E**) Time dependent mechanical alterations of HL60 cells in presence of different surface-functionalized AuNPs. For control condition, same volume of water was added to the cells instead of AuNPs. Data presented is the respective mean value of three independent experimental replicates. Statistical analysis has been done using linear-mixed models. Error bars correspond to the standard error of mean (*p < 0.05; **p < 0.01; ***p < 0.001).

Figure 4A gives a schematic representation of a RT-DC setup while Fig. 4B,C highlight typical scatter plots for deformation and Young’s modulus *vs.* cell size, respectively. Figure 4D,E shows the time dependent alterations of cell deformation and Young’s modulus for HL60 cells in presence of different surface-functionalized AuNPs, respectively. Monitoring the mean cell deformation in protein poor medium over time for three experimental replicates where each measurement consists of several thousand single cell measurements, only a significant decrease in deformation has been observed for dex-10-AuNPs (p = 0.04) after 0.5 h exposure. In contrast, an increase in the mean Young’s modulus was observed for the dex-10-, citrate- and chitosan-AuNPs at a short incubation time (0.5 h) but not at later time points of incubation (4, 15 and 24 h).

We presume that at 0.5 h incubation, except dextrin-AuNPs all other AuNPs form aggregates in the protein poor medium (see Fig. 1) which may interrupt the stability of actin filaments and results in stiffening of HL60 cells. With increasing time, AuNPs form stable and spherical aggregates by adsorbing various biomolecules on their surface and have little influence on the cytoskeleton and correspondingly on cell stiffness.

We have further increased the AuNP concentration (50 nM) and studied the Young’s modulus at two different time points (0.5 and 24 h) (Fig. S7). At 0.5 h incubation, all the AuNPs showed insignificant changes in cell deformation as well as Young’s modulus except dex-10-AuNPs. In contrast, we observed a significant decrease in deformation and an increase in cell stiffness in presence of all AuNPs after 24 h. The alterations in cell stiffness could be either due to change in the adsorption behavior of biomolecules on the NPs surface or uptake or aggregation behavior of the NPs. These results suggest that the presence of AuNPs can potentially change the stiffness of HL60 cells. We believe that lower concentration of the AuNPs (50 nM) can be used for drug delivery applications as they very little affect the mechanical behavior of HL60 cells.

**Figure 5:**
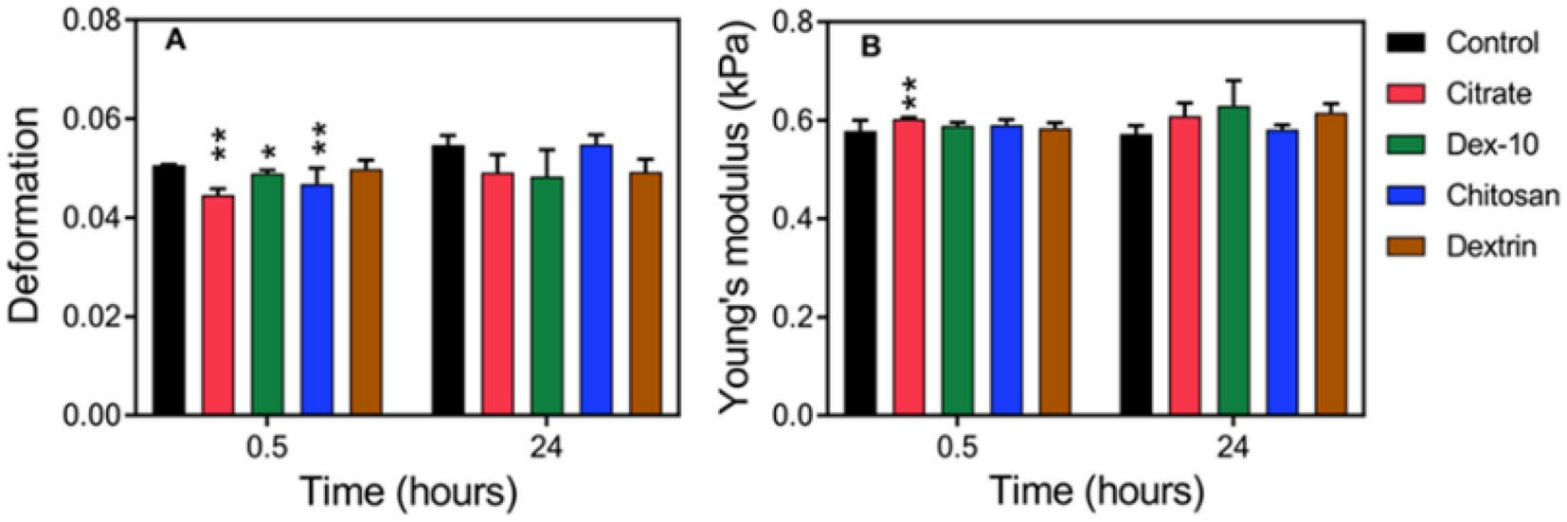
Time dependent mechanical alterations of HL60 cells in presence of different surface-functionalized AuNPs (50 pM) in RPMI with FCS. (**A**) Cell deformation and (**B**) Young’s modulus of HL60 cells incubated with AuNPs. Data presented is the respective mean value of three independent experimental replicates. Statistical analysis has been done using linear-mixed models. Error bars correspond to the standard error of mean. (*p < 0.05; **p < 0.01; ***p < 0.001).

To understand the effect of FCS, similarly, we have studied the mechanical properties of HL60 cells after 0.5 h and 24 h exposure in protein rich media (Fig. 5). We found that except dextrin-AuNPs, all other AuNPs showed a significant decrease in deformation at 0.5 h, whereas no such changes were observed at 24 h. However, only citrate-AuNPs showed increased cell stiffness at 0.5 h, but no such effect was observed at 24 h. Interestingly, after 24 h all AuNPs showed no significant changes in cell deformation and cell stiffness.

### Viability of HL60 cells after treatment with AuNPs at different time intervals

**Figure 6:**
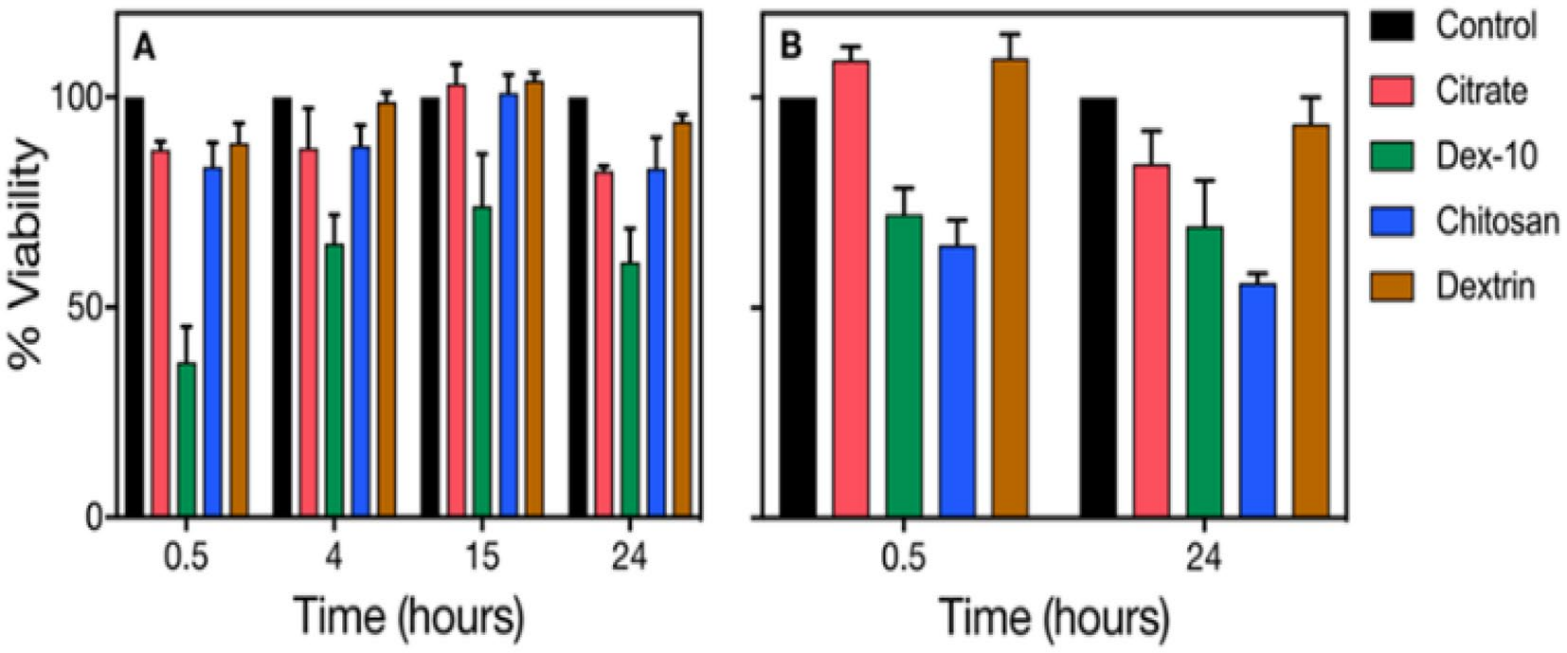
Time dependent cell viability results of HL60 cells in presence of different surface-functionalized AuNP (50 pM) in RPMI media (**A**) without FCS, (**B**) with FCS. Error bars correspond to the standard deviation.

To understand the influence of mechanical stress on the cell viability, we investigated different surface-functionalized AuNPs incubated with HL60 cells in media with and without FCS protein at different time intervals. Figure 6A shows the time dependent cell viability of HL60 cells after treatment with different surface-functionalized AuNPs in media without FCS. Stronger luminescence signal indicates higher amounts of vital cells. AuNPs without FCS can avoid any unspecific interactions with the cells. Except dex-10-AuNPs, all other studied AuNPs maintained a significant cell viability until 15 h, while a slight decrease was found after 24 h. The decreased cell viability for dex-10-AuNPs might be due to the formation of non-spherical aggregates which are more toxic to the HL60 cells. However, there was no change in the Young’s modulus after 4 h incubation at lower concentration.

We have increased AuNP concentration (50 nM) to understand the effect of concentration on cell viability in protein poor medium at different time intervals (Fig. S8). Except dextrin-AuNPs, all other types of surface-functionalized AuNPs showed decreased cell viability at all time points. The increased cell viability for dextrin-AuNPs compared to the other AuNPs is due to the formation of stable bioconjugates which are less toxic to the HL60 cells than spherical and non-spherical aggregates. Both citrate- and dex-10-AuNPs showed decreased cell viability until 15 h, but a slight increase in cell viability for dex-10-AuNPs was observed for 24 h. The decrease in cell viability might be due to the formation of spherical and non-spherical aggregates. However, chitosan-AuNPs showed increased viability with time (max at 15 h and 24 h). The decrease in cell viability for chitosan-AuNPs at shorter incubation (0.5 h) might be due to the formation of spherical aggregates. However, all studied AuNPs at higher concentration exhibited an increase in the Young’s modulus after 24 h incubation.

Figure 6B shows the cell viability after treatment with different surface-functionalized AuNPs in media with FCS, at two different time points (0.5 and 24 h). Interestingly, both dex-10, chitosan-AuNPs showed a substantial decrease in viability at both time points, whereas dextrin-AuNPs showed increased viability at both time points. Citrate-AuNPs showed a slight decrease in cell viability at 24 h. However, no significant change was observed in the Young’s modulus after 24 h incubation in protein rich CCM.

Among all AuNPs, dextrin-AuNPs showed increased viability in both protein poor- and rich medium. Dex-10-AuNPs showed decreased viability in both protein poor- and rich medium. It is quite interesting that chitosan-AuNPs showed increased cell viability in protein poor medium than protein rich medium. Instead, citrate-AuNPs showed increased cell viability in protein rich medium compared to protein poor medium. Our results suggest that there is no correlation between cell stiffness and cell viability. Increase in the cell stiffness might be due to the rearrangement of actin and tubulin microfilaments by the AuNPs, whereas decrease in cell viability might be due to the formation of aggregates and their uptake.

### Release of ROS upon interaction with AuNP

Finally, we have investigated the release of reactive oxygen species (ROS) in HL60 cells incubated with AuNPs in both protein poor- and rich CCM. ROS are chemically reactive molecules, and can promote cell proliferation and differentiation at a moderate level. However, an elevated level of ROS can induce oxidative stress, resulting in severe damage to the DNA, protein, and cells^55,56^. It was previously reported that upon interaction with NPs, intracellular ROS production may increase and by interfering with cellular organelles can cause DNA/RNA breakage, membrane destruction and increased toxicity and eventually cellular death^57^.

**Figure 7:**
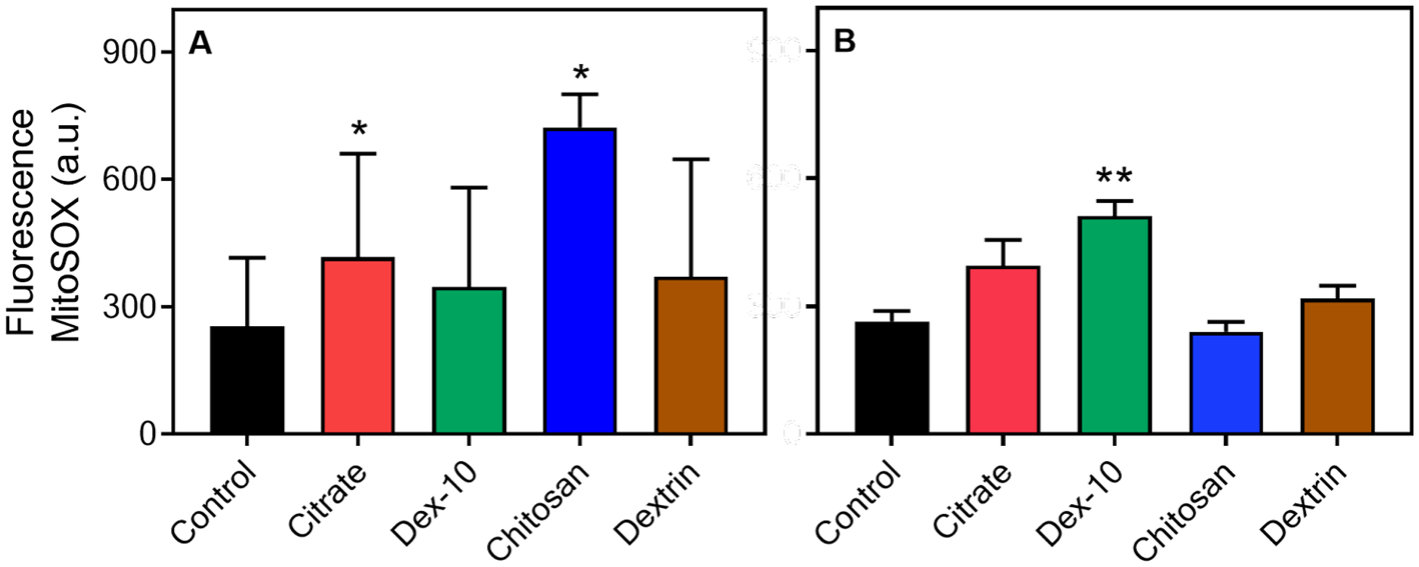
ROS production caused by various surface-functionalized AuNPs (50 pM) in surrounding media and HL60 cells. Cells were incubated with AuNPs for 24 h in RPMI medium without FCS (**A**) and with FCS (**B**) as well as both were treated with MitoSOX-red fluorescence based mitochondrial superoxide indicator. Real-time fluorescence and deformability cytometry were used to assess the fluorescence levels. (*p < 0.05; **p < 0.01).

Mitochondria are important source of ROS in a cell. MitoSOX-red was used as a mitochondrial ROS (superoxide) indicator to investigate whether NPs stimulate excess of ROS release. Figure 7A,B shows the release of ROS in HL60 cells induced by different surface-functionalized AuNPs (50 pM) in protein poor- and rich CCM, respectively. In case of protein poor media, both positively charged chitosan-AuNPs (p = 0.04) and negatively charged citrate-AuNPs (p = 0.01) produced more ROS than incubation with neutral dxt/dex-10-AuNPs. This could be either due to the aggregation of AuNPs or due to a strong electrostatic interaction between AuNPs and the cellular components. Among all the AuNPs in protein rich media, dex-10-AuNPs showed a significant increase (p = 0.002**) in the ROS levels. Citrate-AuNPs induced moderate increase in the ROS levels, whereas chitosan-, dextrin-AuNPs showed almost no influence on the ROS levels. Even though some reports suggest that chitosan triggers ROS induction in cancer cells, we did not observe any difference in the ROS levels in HL60 cells; this might be due to lower concentration of AuNPs^58^. A moderate increase of ROS levels can act as a second messenger for physiological activities, however excessive ROS levels may increase the antioxidant capacity of cell and trigger cell death^59^.

## Conclusion

In this work we have investigated the time-dependent stability of different surface-functionalized AuNPs in protein poor- and protein rich CCM and their effect on the cell physiology and cell viability. AuNPs were functionalized with various ligands i.e. citrate, dex-10, dextrin and chitosan. We found that the formation of AuNP-biomolecular conjugates dependents on both nanoparticle surface and CCM medium. Both UV–Vis and DLS analyses showed a slow development of AuNP-biomolecular aggregated conjugates in protein poor CCM (citrate, dex-10), whereas stable conjugates were observed in protein rich CCM. Especially, dextrin-AuNPs showed stable conjugates in both media, whereas chitosan-AuNPs showed both stable and spherical aggregates in both media. We found that the stiffness and viability of the cells by the AuNPs alters with incubation time, surface functionality, NP concentration, aggregation behaviour and CCM. Dextrin-AuNPs formed stable bioconjugates in both CCM with little impact on cell stiffness, ROS production and cell viability. Chitosan-AuNPs induced significant changes in stiffness in protein poor-CCM at shorter incubation time (0.5 h) and showed increased cytotoxicity in protein rich CCM with increased ROS levels in protein poor CCM. Dex-10-AuNPs lead to significant changes in cell stiffness in protein poor medium at short incubation time (0.5 h) and showed increased toxicity in both CCM with increased ROS levels in protein rich CCM. Incubation with citrate-AuNP bioconjugates yielded a significant increase in cell stiffness at shorter incubation times (0.5 h) and insignificant changes in cell viability in both CCM with moderate ROS levels in protein poor- and rich medium. Our results demonstrate that dextrin-AuNPs could be used as a safe material for biomedical applications as they showed lower toxicity and insignificant changes in cell physiology. In addition, the implementation of the RT-DC technique allows characterizing with high-throughput the mechanical properties of cells upon interaction with nanoparticle candidates for biomedical applications.

## Experimental sections

### Materials

Tetrachloroauric acid (HAuCl_4_) and dextrin were purchased from Sigma-Aldrich (Taufkirchen, Germany). Chitosan, dextran-10, trisodium citrate, EtOH, HCl and NaOH were purchased from Roth (Karlsruhe, Germany). All chemicals were used as received. The water used was purified through an ultrapure water system, Millipore system and Sartorius Stedim Biotech (Göttingen, Germany).

### Synthesis of citrate-AuNPs

Citrate-AuNPs were synthesized according to the standard Turkevich method^60^. Briefly, a solution of HAuCl_4_ (500 mL, 0.5 mM) was heated up to boiling in an 1 L Erlenmeyer flask, followed by the slow addition of trisodium citrate solution (25 mL, 1% w/v) under vigorous stirring. After 15 min of boiling, the reaction was cooled to room temperature and stored at 4 °C for further use.

### Synthesis of chitosan-AuNPs

0.125 M HAuCl_4_ aqueous solution was added to the 0.2 w/v% chitosan solution (1% acetic acid) under boiling temperature and allow the stirring for 30 min or until the color of the solution changed from colorless to ruby red. Then, the mixture was allowed to cool to room temperature and centrifuged (4 °C, 15 × *g* for 30 min) and redispersed in 20 mL deionized water.

### Synthesis of dextran-10 and dextrin-AuNPs

19 mM HAuCl_4_ solution was added to 30 mL of dextran-10 (1 w/v %) aqueous solution under stirring at RT. After 30 min of stirring, 0.05 M NaOH was added to the above solution until color changed from yellow to colorless. Next, the solution was boiled for 30 min and allowed the mixture to cool to RT. Then the mixture was centrifuged (4 °C, 20 × *g* for 30 min) and the pellet was redispersed in 20 mL deionized water. Similar procedure was used for the dextrin-AuNPs.

### Sample preparation

The stability of different surface-functionalized AuNPs in CCM at 37 ℃ was analyzed for different incubation periods (0.5, 4, 15 and 24 h). We used two different CCM (with and without 10% FCS) mixed with different surface-functionalized AuNPs. RPMI medium with 10% FCS was referred as protein rich medium whereas RPMI without FCS referred as protein poor medium. The concentration of all the AuNPs in the CCM was 100 nM, and the AuNPs were incubated in Eppendorf tubes until they were analyzed.

### UV–Vis absorption spectroscopy

UV–Vis spectra of different surface-functionalized AuNPs and the same in RPMI protein poor- and rich medium were measured using NanoDrop 2000c spectrophotometer (Thermo Scientific, Germany) in a 10 mm path length cuvette (Brand UV cuvettes, Germany) at 25 ℃. The spectra were recorded between 200 and 800 nm.

### Dynamic light scattering (DLS) and zeta potential measurements

The hydrodynamic diameter (d_H_) and the zeta potential for different surface-functionalized AuNPs and the same in RPMI protein poor- and rich medium were measured using a Zetasizer Nano-ZS (Malvern Instruments, Herrenberg, Germany). Samples were prepared as described above and filtered through a 0.2 μm (for AuNPs) filter followed by equilibration (typically 5 min) at 25 °C. The AuNPs in CCM was measured without filtration. Measurements were taken at a detector angle of 173° using a refractive index of 1.33 and 1.44 for water and CCM, respectively. Five independent measurements (12 runs per measurement with a run duration of 5 s) were carried out to estimate d_H_ of the samples, with measurement uncertainty indicated as standard deviation. The zeta potential measurements were carried out at 25 °C with 5 min equilibration between each measurement and a voltage of 180 V (for water) and 40 V (for CCM) using disposal DTS1070 cuvettes. The reported zeta potential is an average of five independent measurements of 20 runs (each with duration of 5 s).

### Osmolarity measurements

Osmolarity of both protein poor- and rich RPMI medium was measured using an Osmometer (Fiske micro-Osmometer, model 210, Fiske associates-PA, USA) as 290 mOsm at two different time points. All the surface-functionalized AuNPs were added to the both protein poor- and protein rich CCM and incubated for 0.5 h and 24 h at 37 ℃ and 5% CO_2_. Osmolarity was measured at the end of each time point and the results were plotted using GraphPad Prism 7 (GraphPad Software).

### Transmission Electron Microscopy (TEM)

AuNPs samples (4 µL) were blotted on carbon-coated 400 square mesh copper grids and then air-dried. TEM micrographs were obtained using a JEOL JEM-1400PLUS transmission electron microscope operating at an acceleration voltage of 120 kV.

### Cell culture

We performed all the experiments on a human myeloid precursor cell line HL60 (courtesy of Don and Ada Olins, University of New England). Cells are cultured in RPMI 1640 medium (Roswell Park Memorial Institute Medium no. 1640, Biowest) along with 10% FCS (FCS, Gibco, ThermoFisher Scientific, Massachusetts, USA), 1% penicillin/streptomycin (BioWest) and 2 mM l-glutamine (BioWest) at 37 ℃ and 5% CO_2_. Cells are passaged every 48 h by harvesting and resuspension to approximately 0.2 million cells/mL of fresh medium. Viability of 95% is assessed using trypan blue, and all the experiments are carried out approximately 36 h after passage (when the cells are in log phase). Cells are harvested by centrifugation at 200× RCF for 5 min (Allegra X-15R, Beckmann Coulter), and around 1 × 10^6^ cells/mL are used for each measurement. Cell cultures are tested to be mycoplasma free regularly.

### Real-time deformability cytometry (RT-DC)

RT-DC is used for high-throughput cell mechanical characterization based on the AcCellerator (Zellmechanik Dresden)^39–41^. The system consists of an inverted microscope and a CMOS camera, which captures the images of cells passing through the microfluidic chip assembled on a xy-stage. Two syringes provide sheath and sample flow at dedicated flow rates. Cells translocate through a constriction of 300 µm length and 30 µm × 30 µm cross-section and deform by hydrodynamic shear and normal stress. Image acquisition and analysis are performed in real-time with a throughput exceeding 1000 cells per s and deformation is quantified using the circularity of each particle:

$$Deformation=1-circularity=1-\frac{2\surd \pi Area}{perimeter}$$

The elastic modulus of cells is obtained from deformation and cell size applying a model published earlier^40,41^.

### Acquisition and analysis of RT-DC data

The sample consisted of cells after NPs treatment (see sample preparation) harvested by centrifugation at 200× RCF for 5 min resuspended in PBS (without Ca^2+^/Mg^2+^) and complemented with Methylcellulose at 0.5% (w/v). This Cell Carrier A buffer (CCA, Zellmechanik Dresden) was injected in the microfluidic chip at a flow rate of 0.16 µL/s and constantly maintained during each measurement. Brightfield images of individual cells were recorded using ShapeIn (version 2.0.5, Zellmechanik Dresden). In an experiment, approximately 10,000 single-cell events were captured. The statistical analysis was performed in ShapeOut (version 0.8.4, Zellmechanik Dresden) by applying an area-ratio filter of 1.05 to account for a 5% maximum tolerance in deviation of the convex hull area from the cell area. An additional area filter 50–400 µm^2^ was used to excluded small debris particles and cell clusters. For statistical analysis, a pairwise comparison between two groups was conducted using linear mixed models on a single cell level^61^. Differences in an observable, such as the Young’s modulus, are assigned to random and fixed effects, respectively. Random effects account for systematic and stochastic measurement biases, such as variable different NPs treatment. The amplitude or fold change of an experimental quantity is represented by fixed effects. The maximum likelihoods are determined after calculating statistical significance using two models, one with and the other without fixed effects. The associated p-values are calculated using the likelihood ratio and Wilks' theorem. The results were plotted using GraphPad Prism 7 (GraphPad Software).

### Cytotoxicity assay

Cell viability was assessed by the CellTiter-Glo 2.0 assay from Promega (Madison, USA) following manufacturer’s instructions. Briefly, 5 × 10^4^ cells/mL were seeded in RPMI media with and without FCS according to the treatment. The cells were cultured for 24 h prior to the treatment and they are incubated with AuNPs at different time points (0.5, 4, 15 and 24 h respectively) in a 96 well opaque walled plate at 37 °C and 5% CO_2_. After respective time points the cells were added with 100 µL of CellTiter-Glo 2.0 and incubated for 10 min before proceeded for the measurement. The cells were measured for the luminescence signal using a SpectraMax Paradigm (Molecular Devices, San Jose, USA). The viability of the cells was assessed by plotting the graph for the intensities, by normalizing to control at different time points for each treatment condition after background subtraction.

### ROS detection by real-time fluorescence and deformability cytometry (RT-FDC) and MitoSOX

RT-FDC is an advancement of RT-DC and enables the simultaneous analysis of cell mechanical properties and fluorescence emission (Zellmechanik Dresden)^62^. Sample measurement was carried out as above (see section RT-DC). RT-FDC was used to analyze levels of ROS produced in the cells upon interaction with AuNPs. We have analyzed ROS produced in the cells upon interaction with AuNPs, where MitoSOX-red (ThermoFisher Scientific) has been used as an indicated for mitochondrial superoxide (Ex/Em: 510/580 nm). Cells were treated with AuNPs and incubated in medium with, and without serum for 24 h. After 24 h, the cells were treated with 1 µM MitoSOX-red (ThermoFisher Scientific) for 10 min. The cells were then collected, centrifuged (200× RCF for 5 min) to remove the supernatant and the pellet was resuspended in CCA before proceeding with the measurement. The measurement was done by ShapeIn (version 2.0.5, Zellmechanik Dresden) and analysis was done by ShapeOut (version 0.8.4, Zellmechanik Dresden).

## Supplementary Information

Supplementary Figures.

## Data Availability

The data that support the findings of this study is available from the corresponding author upon reasonable request. The absorption and size distribution analyses are available in Origin files. The raw RT-DC and RT-FDC data are available as TDMS files that can be read, processed, and analyzed by ShapeOut.
