# Supplementary material for: The impact of cell culture media on the interaction of biopolymer-functionalized gold nanoparticles with cells: mechanical and toxicological properties
Source: Sci Rep. 2022 Oct 5;12:16643. doi: 10.1038/s41598-022-20691-w (PMC9534915; doi:10.1038/s41598-022-20691-w)
Supplement: Supplementary file 1 — Supplementary Figures. [file 41598_2022_20691_MOESM1_ESM.docx]

Supporting Information

**The impact of cell culture media on the interaction of biopolymer-functionalized gold nanoparticles with cells: mechanical and toxicological properties**

Brahmaiah Meesaragandla,^1,2^ Yesaswini Komaragiri,^2,3,4^ Rabea Schlüter,^5^ Oliver Otto,^2,3,4^ and Mihaela Delcea^1,2,3^*

*^1^Biophysical Chemistry, Institute of Biochemistry, University of Greifswald, Felix-Hausdorff-Straße 4, 17489 Greifswald, Germany*

*^2^ZIK HIKE - Zentrum für Innovationskompetenz „Humorale Immunreaktionen bei kardiovaskulären Erkrankungen“, Fleischmannstraße 42, 17489 Greifswald, Germany*

*^3^DZHK (Deutsches Zentrum für Herz-Kreislauf-Forschung), partner site Greifswald, Germany*

*^4^ Institute of Physics, University of Greifswald, Felix-Hausdorff-Strasse 6, 17489 Greifswald, Germany*

*^5^ Imaging Center of the Department of Biology, University of Greifswald, Friedrich-Ludwig-Jahn-Str. 15, 17489 Greifswald, Germany.*

*Corresponding author, [delceam@uni-greifswald.de](mailto:delceam@uni-greifswald.de)

**Figure S1**. Molecular structures of ligands.

**Figure S2**. UV-Vis absorption spectra of different surface-functionalized AuNPs.

**Figure S3**. Transmission electron micrographs of AuNPs functionalized with citrate, dex-10, chitosan and dextrin molecules.

**Figure S4**. DLS and zeta potential data of AuNPs functionalized with citrate, dex-10, dextrin and chitosan ligands.

**Figure S5**. Zeta potential data of AuNPs in protein poor- and protein rich medium.

**Figure S6**. Osmolarity of protein poor- and protein rich CCM with different surface-functionalized AuNPs.

**Figure S7.** Time-dependent mechanical alterations of HL 60 cells in presence of AuNPs in RPMI without FCS.

**Figure S8.** Time dependent cytotoxicity results of HL 60 cells in presence of different surface-functionalized AuNP in RPMI media without FCS.


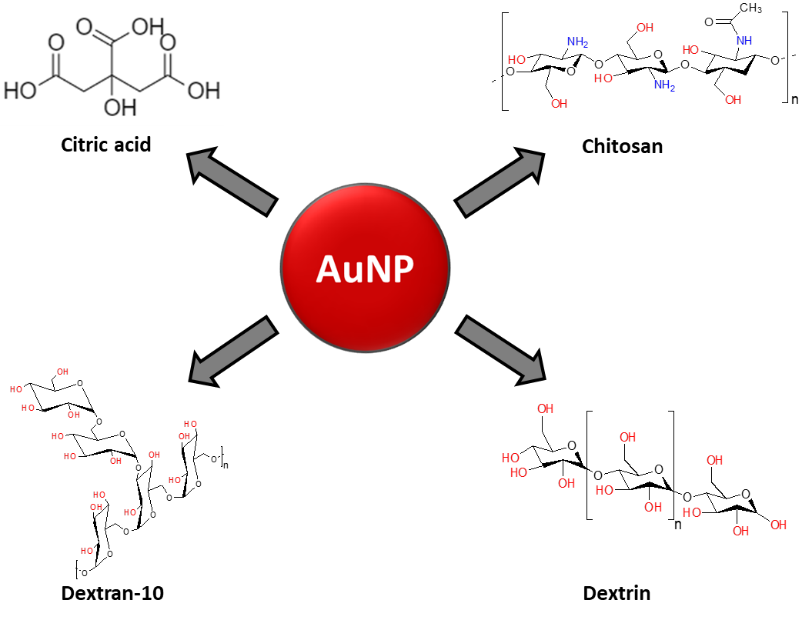


**Figure S1.** Molecular structures of ligands (drown with ChemDraw) used to functionalize the AuNPs.


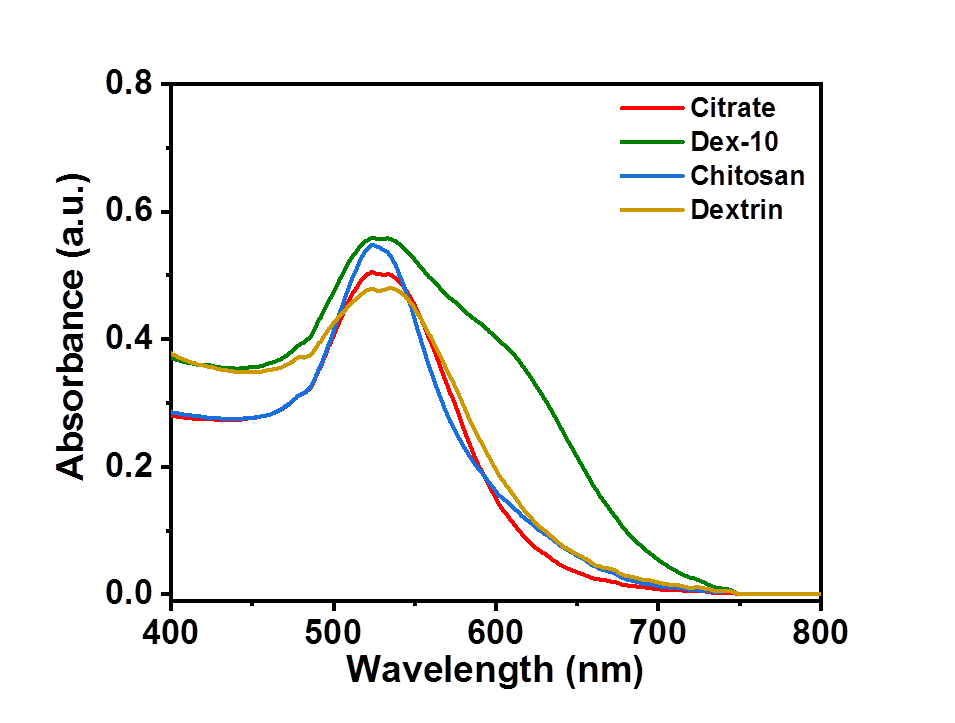


**Figure S2.** UV-Vis absorption spectra of AuNPs functionalized with citrate, dex-10, chitosan and dextrin ligands (AuNPs concentration = 100 nM).


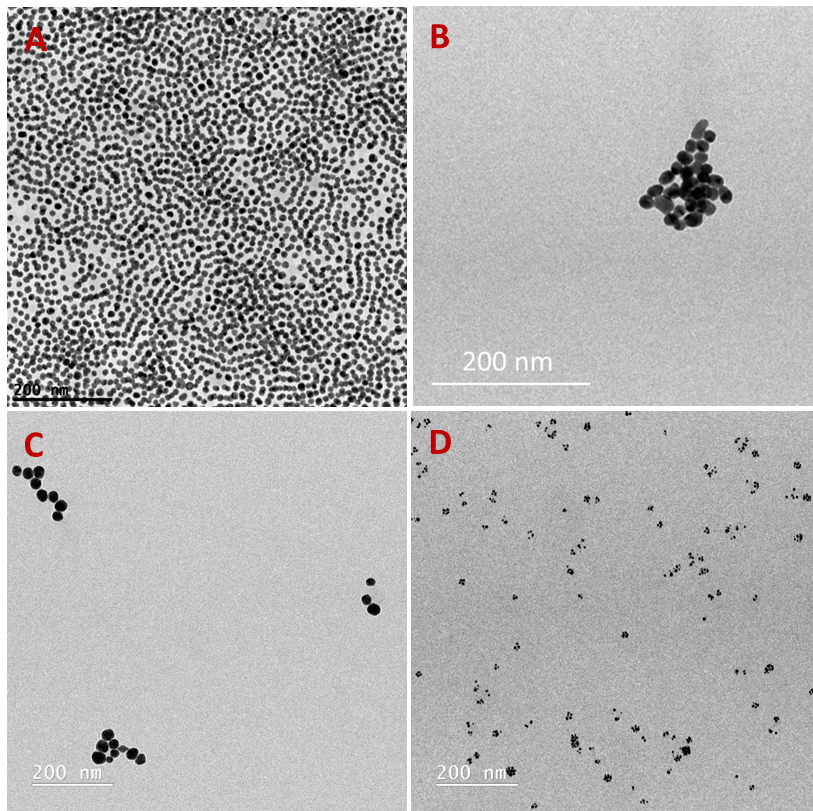


**Figure S3.** Transmission electron micrographs of AuNPs functionalized with citrate (A), dex-10 (B), chitosan (C) and dextrin (D) ligands.


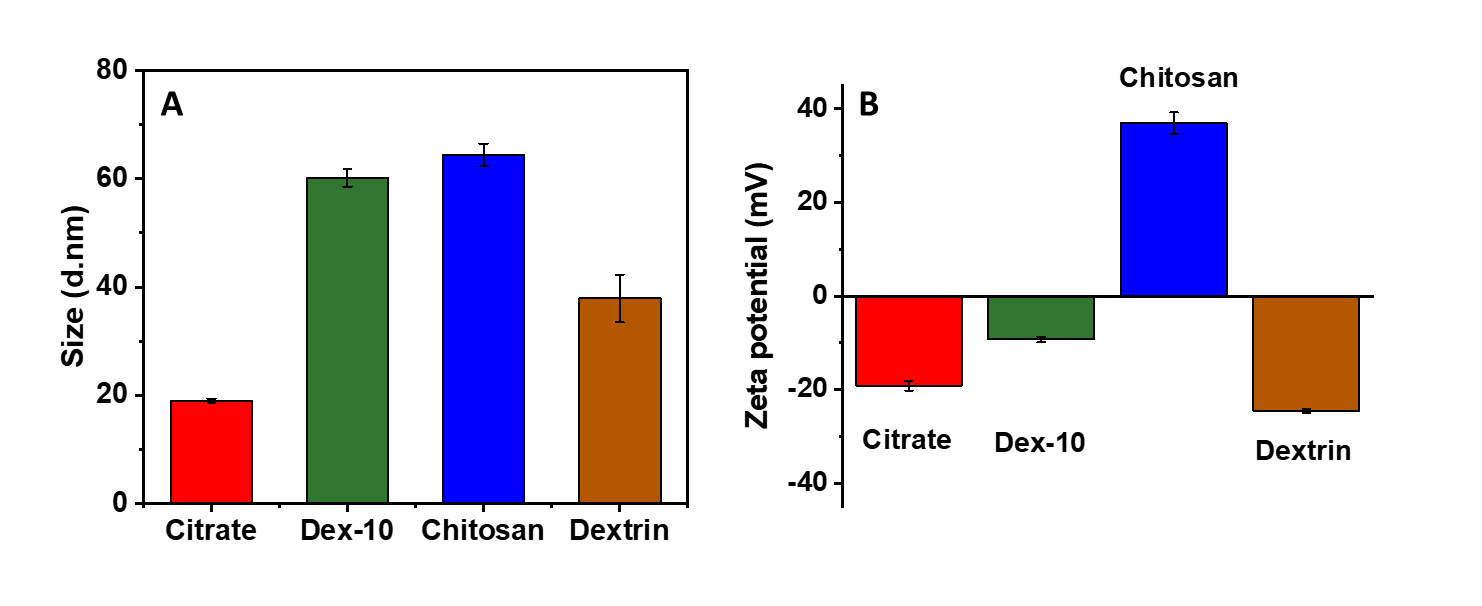


**Figure S4.** DLS (A) and zeta potential (B) data of biopolymer-functionalized AuNPs.

**
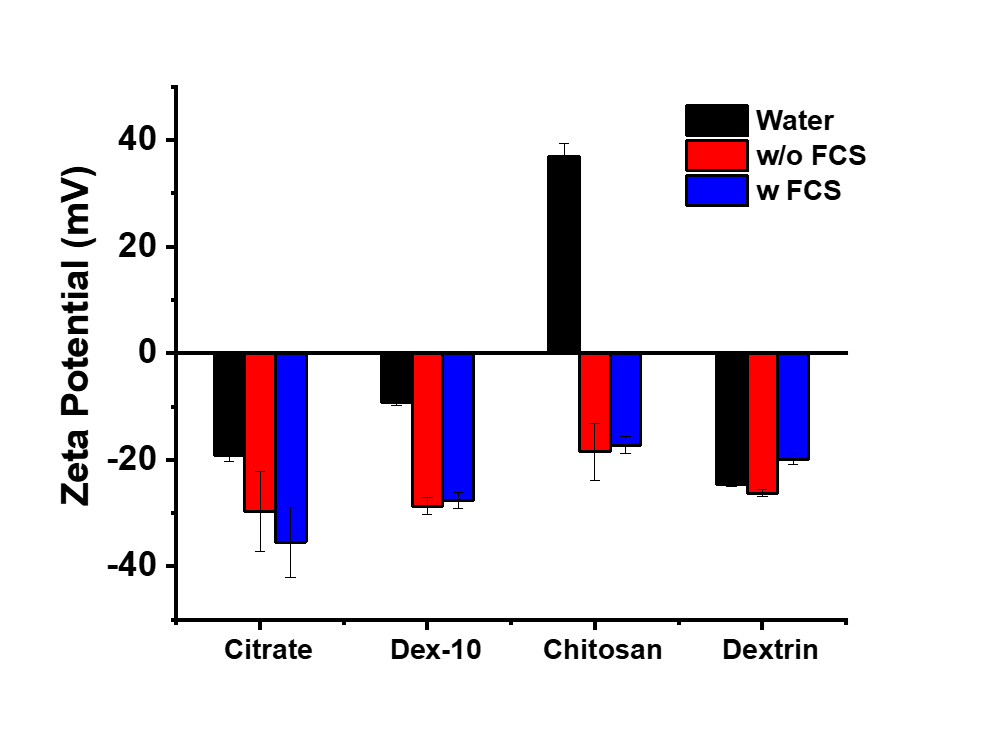
**

**Figure S5.** Charge distribution of different surface-functionalized AuNPs in protein poor- and rich media at 37 ºC after 24 h incubation.


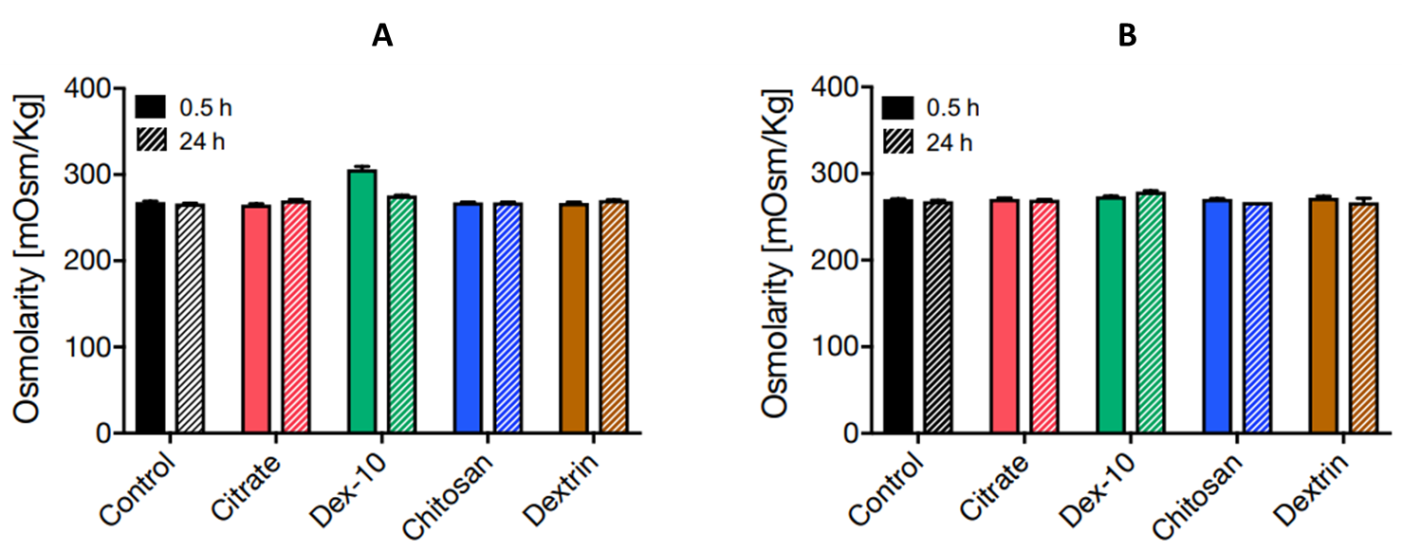


**Figure S6.** Osmolarity of protein poor (A) and protein rich (B) CCM after incubation with different surface-functionalized AuNPs for 0.5 h and 24 h at 37 ºC.


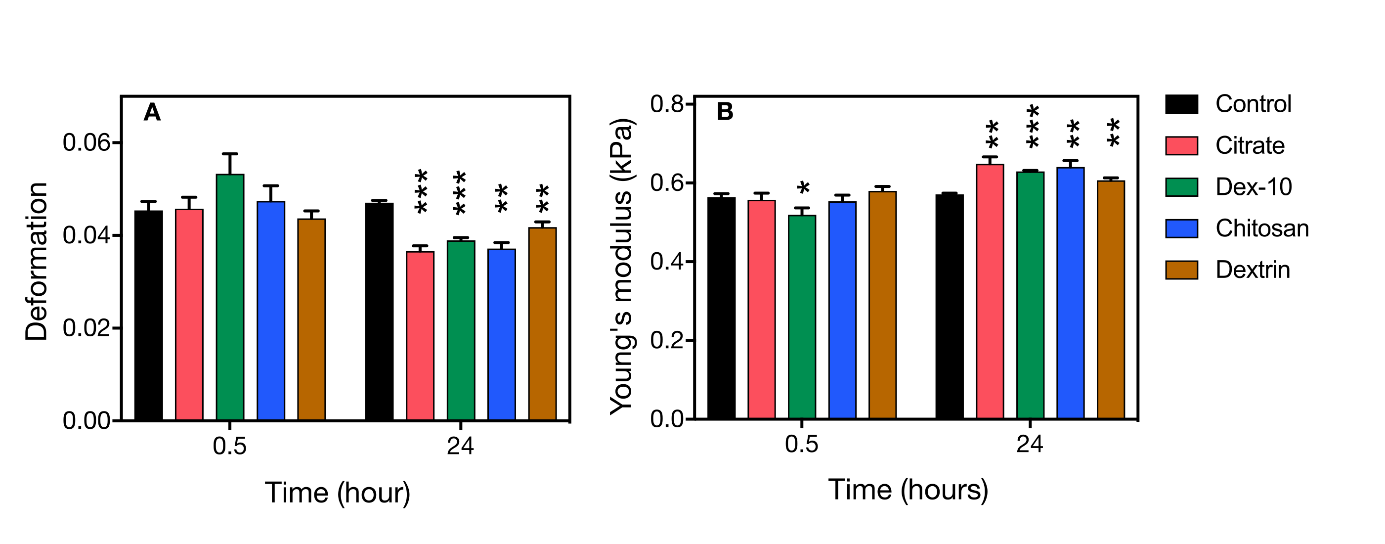


**Figure S7.** Time-dependent mechanical alterations of HL60 cells in presence of AuNPs (50 nM) in RPMI without FCS. Error bars correspond to the standard error of mean.

**
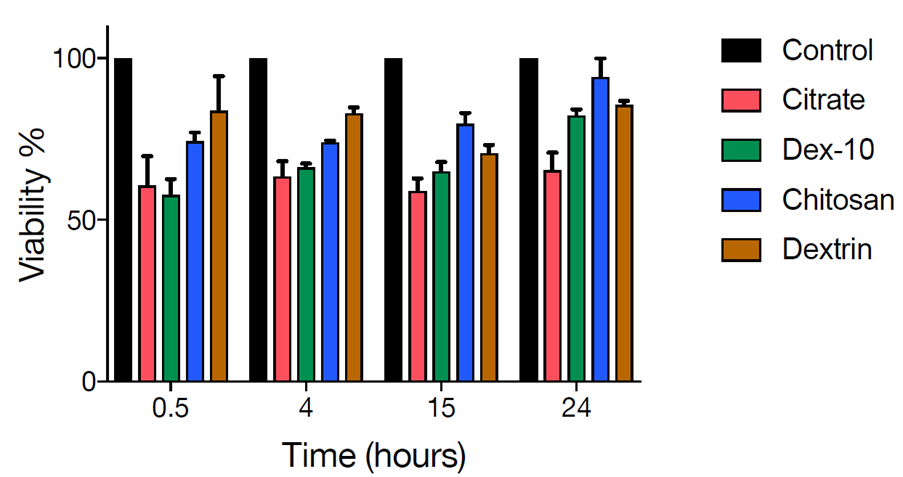
**

**Figure S8.** Time dependent cytotoxicity results of HL 60 cells in presence of different surface-functionalized AuNP (50 nM) in RPMI media without FCS. Error bars correspond to the standard deviation.
